# Supplementary material for: Enhancing Erythropoiesis by a Phytoestrogen Diarylheptanoid from Curcuma comosa
Source: Biomedicines. 2022 Jun 16;10(6):1427. doi: 10.3390/biomedicines10061427 (PMC9219836; doi:10.3390/biomedicines10061427)
Supplement: Supplementary file 1 [file biomedicines-10-01427-s001.zip › biomedicines-1738355-supplementary.pdf]

Supplementary materials

Enhancing erythropoiesis by a phytoestrogen diarylheptanoid from *Curcuma comosa*

Kanit Bhukhai[1,*], Guillemette Fouquet[2,3], Yutthana Rittavee[4], Nopmullee Tanhuad[1], Chaiyaporn Lakmuang[5], Suparerk Borwornpinyo[6,7], Usanarat Anurathapan[8], Apichart Suksamrarn[9], Pawinee Piyachaturawat[1], Arthit Chairoungdua[1,7], Olivier Hermine[2,3,10,11], and Suradej Hongeng[8,*]

[1]Department of Physiology, Faculty of Science, Mahidol University, Bangkok, Thailand
[2]Institut Hospitalo-Universitaire Imagine, Université Sorbonne Paris Cité, Assistance Publique-Hôpitaux de Paris, Hôpital Necker, Paris, France
[3]INSERM U1163 and CNRS ERL8254, Université Paris Descartes, Faculté de Médecine, Hôpital Necker, Par-is, France
[4]Department of Biology, Faculty of Science, Mahidol University, Bangkok, Thailand
[5]Department of Chemistry, Faculty of Science, Mahidol University, Bangkok, Thailand
[6]Department of Biotechnology, Faculty of Science, Mahidol University, Bangkok, Thailand
[7]Excellent Center for Drug Discovery, Mahidol University, Bangkok, Thailand
[8]Department of Pediatrics, Faculty of Medicine, Ramathibodi Hospital, Mahidol University, Bangkok, Thailand
[9]Department of Chemistry and Center of Excellence for Innovation in Chemistry, Faculty of Science, Ram-khamhaeng University, Bangkok, Thailand
[10]Laboratory of Excellence GReX, Paris, France
[11]Service d'Hématologie Clinique Adultes, Assistance Publique-Hôpitaux de Paris, Hôpital Necker, Paris, France

*Correspondence: Kanit Bhukhai, email: kanit.bhu@mahidol.ac.th, Tel.: +6622015614
Suradej Hongeng, email: suradej.hon@mahidol.ac.th, Tel.: +6622011495

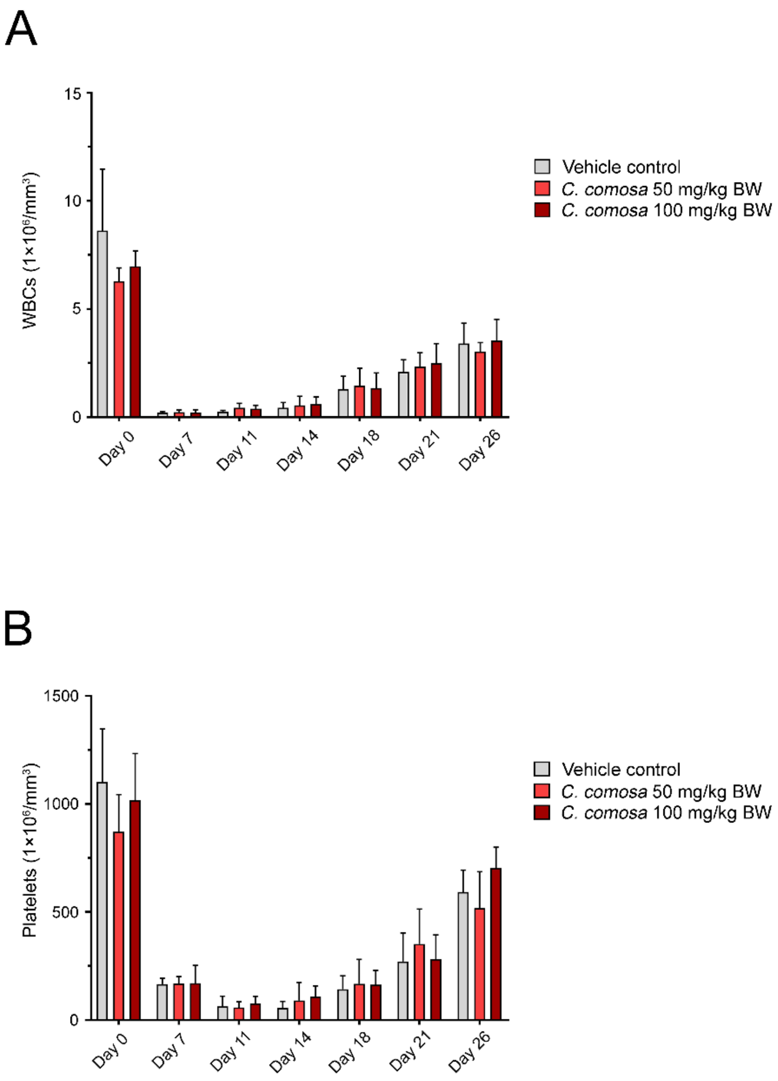

Figure S1. Effects of *Curcuma comosa* extract on WBCs and platelets in anemic mice. Hematologic response of mice to sublethal irradiation induced-anemia: **(A)** WBC and **(B)** platelet counts at days 7, 11, 14, 18, 21, and 26. Irradiated mice were intraperitoneally injected with vehicle or *C. comosa* extract (50 or 100 mg/kg BW) each day. Blood was collected from the retro-orbital plexus, and hematological parameters were monitored with an MS9 analyzer. All data are expressed as mean ± standard error (n = 10). **P < 0.01, ASPP 049 compared with vehicle control treatment (ANOVA); #P < 0.05 and ##P < 0.01, E2 compared with vehicle control treatment (ANOVA). WBC, white blood cell.

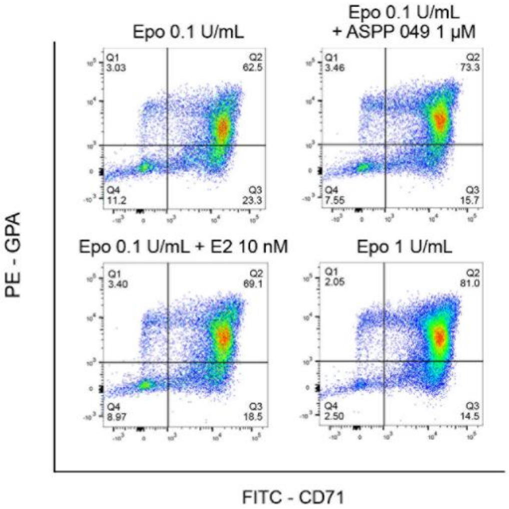

Figure S2. Representative flow cytometry showing effects of ASPP 049 and E2 on immature erythrocyte marker expression. CD34^{+} HSCs plated in human erythroid culture medium were treated with 1 µM ASPP 049 and 10 nM E2 in the presence of 0.1 U/mL of Epo for 14 days; 1 U/mL Epo was used as a positive control. Expression of GPA and CD71 was observed by flow cytometry analysis.

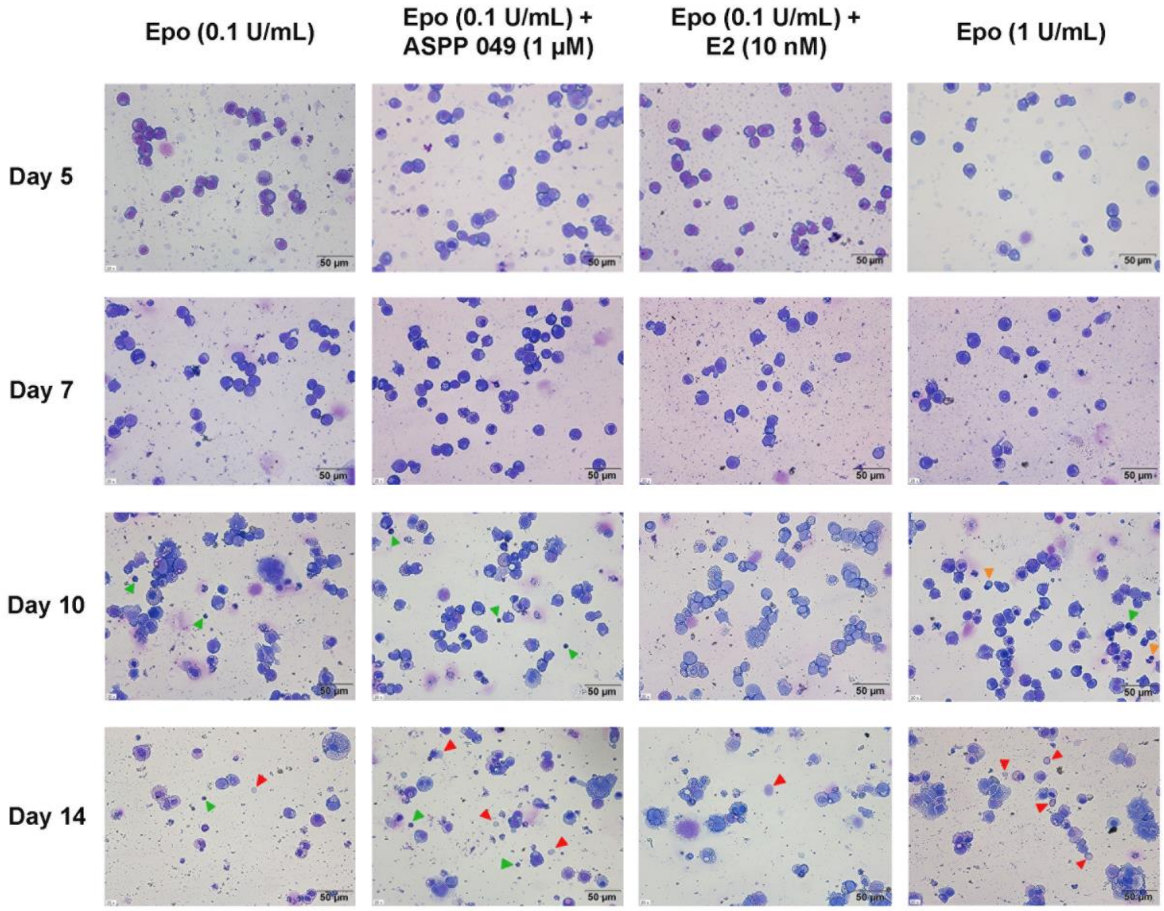

Figure S3. Morphology of erythropoietic response in ASPP 049-treated HSC-derived erythroblasts. CD34$^+$ HSCs plated in human erythroid culture medium were treated with 1 μM ASPP 049 and 10 nM E2 in the presence of 0.1 U/mL of Epo for the indicated times; 1 U/mL Epo was used as a positive control. Morphological characteristics of erythropoietic cells were observed under a microscope using cytospin with Liu staining. E2, 17β-estradiol; Epo, erythropoietin; HSCs, hematopoietic stem cells; green arrowhead, nucleus; orange arrowhead, enucleation; red arrowhead, RBCs.

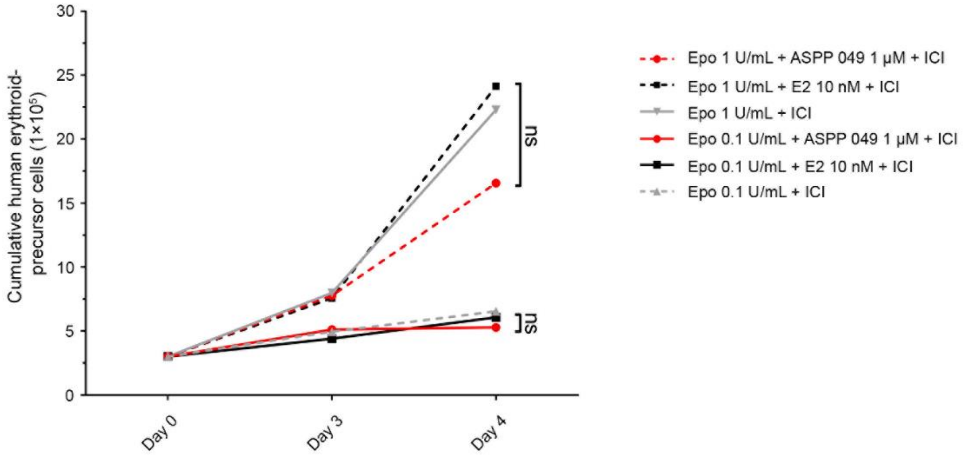

Figure S4. Co-treatment of an ER antagonist with ASPP 049 or E2 failed to enhance erythroid precursor cell proliferation. $CD36^+$ cells sorted from $CD34^+$ HSCs cultured in erythroid medium were treated with 1 μM ASPP 049 and 10 nM E2 in the presence of 0.1 or 1 U/mL of Epo with or without an ER antagonist, ICI 182, 780. Proliferation of $CD36^+$ cells was observed by trypan blue exclusion assay. All data are expressed as mean ± standard error (n = 3). ns, non-significant.

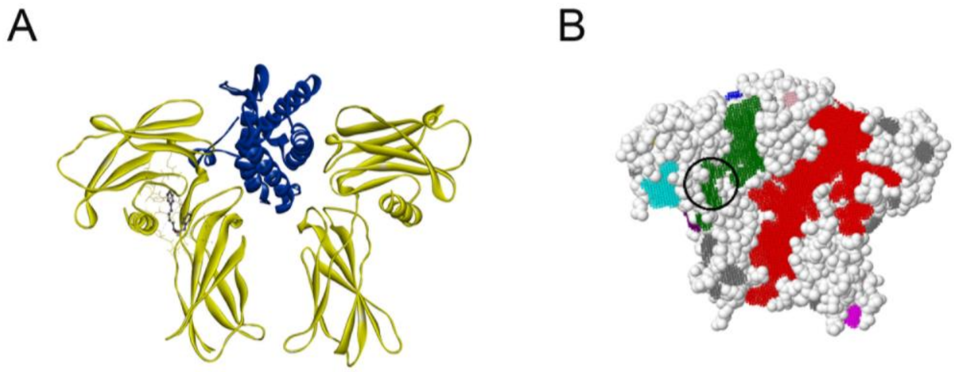

Figure S5. ASPP 049 binds to the Epo-EpoR complex. (A) Lowest energy position of ASPP 049 within the Epo-EpoR complex (crystal structure represented in gold). **(B)** Possible locations for binding site of ASPP 049. Epo, erythropoietin; EpoR, erythropoietin receptor.
